# Glutathione-Triggered Formation of a Fmoc-Protected Short Peptide-Based Supramolecular Hydrogel

**DOI:** 10.1371/journal.pone.0106968

**Published:** 2014-09-15

**Authors:** Yang Shi, Jingyu Wang, Huaimin Wang, Yanhui Hu, Xuemei Chen, Zhimou Yang

**Affiliations:** 1 State Key Laboratory of Medicinal Chemical Biology, Key Laboratory of Bioactive Materials, Ministry of Education, College of Life Sciences, and Collaborative Innovation Center of Chemical Science and Engineering (Tianjin), Nankai University, Tianjin, P. R. China; 2 School of Chemical and Materials Engineering, Hubei Institute of Technology, Huangshi, Hubei, P. R. China; Texas A&M University, United States of America

## Abstract

A biocompatible method of glutathione (GSH) catalyzed disulfide bond reduction was used to form Fmoc-short peptide-based supramolecular hydrogels. The hydrogels could form in both buffer solution and cell culture medium containing 10% of Fetal Bovine Serum (FBS) within minutes. The hydrogel was characterized by rheology, transmission electron microscopy, and fluorescence emission spectra. Their potential in three dimensional (3D) cell culture was evaluated and the results indicated that the gel with a low concentration of the peptide (0.1 wt%) was suitable for 3D cell culture of 3T3 cells. This study provides an alternative candidate of supramolecular hydrogel for 3D cell culture and cell delivery.

## Introduction

Supramolecular hydrogels of peptides have attracted extensive research interests in recent years due to the ease of design and synthesis, easy integration of bioactive ligands, and functionality [1]–[9]. They have shown big potential in three dimensional (3D) cell culture [10], [11], controllable delivery of therapeutic agents [12]–[15], immune boosting [16], sensing [17]–[20], and regenerative medicine [21], [22]. Among the reported peptide hydrogel systems, RADA16 and its derivatives [23], [24], peptide amphiphiles [25], [26], Q11 and its derivatives [27], [28], FEK8 and its analogues [29], [30], and those based on dipeptide of FF [31]–[35] are the most widely investigated. These peptide hydrogels are usually formed by ionic strength or pH change or organic solvent assistance. For the biomedical applications, methods to prepare peptide hydrogels in mild and biocompatible ways will be beneficial, which is actively explored recently [36]. Several biocompatible methodologies have been developed such as enzyme triggeration [37], [38], photo-irradiation [39], [40], redox control [41]–[43], and ligand-receptor interaction promotion [44]. Recently, Nilsson group had firstly developed a novel method of disulfide bond reduction to trigger a supramolecular hydrogel formation [45], which was followed by us to prepare short peptide-based hydrogels for cell culture and drug delivery [46]. We opted to applied this method to prepare hydrogels based on the Fmoc-FF and tested the possibility of the formed hydrogel in 3D cell culture, which was reported in this study.

## Results and Discussion

### Syntheses and rationale of the design

Hydrogels of Fmoc-FF was firstly reported by Ulijn and Gazit groups and the hydrogels have been applied for 3D cell culture [47], [48]. The hydrogels were initially prepared by organic solvent (DMSO) assistance, and therefore a cell culture medium exchange procedure was needed to assist cell growth. Ulijn and co-workers then utilized the enzymatic reaction by a protease to prepare hydrogels based on Fmoc-FF [49].

**Figure 1:**
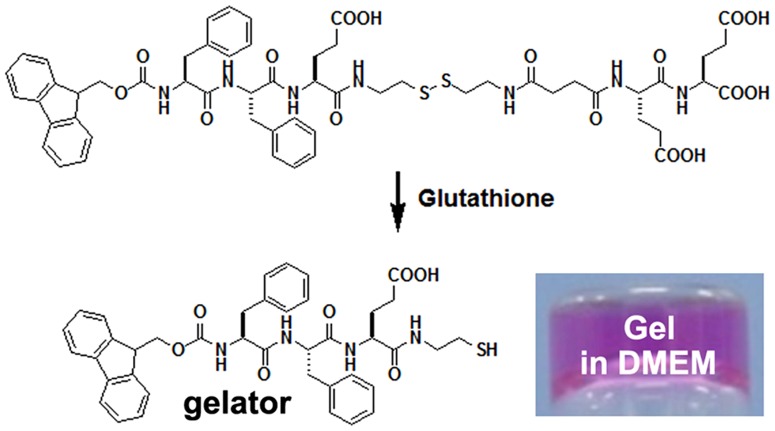
Chemical structure of the pro-gelator (Fmoc-FFE-ss-EE) and the resulting gelator (Fmoc-FFE-s) and an optical image of a gel formed by treating a DMEM solution containing 0.3 wt% of the pro-gelator with 4 equiv. of GSH.

We recently reported on a biocompatible triggeration of disulfide bond reduction catalyzed by glutathione (GSH) to form hydrogels for cell culture [50]. Due to the ease of synthesis of short peptides based on Fmoc-FF and the good properties of hydrogels of Fmoc-FF, we therefore attempted to further apply our GSH-triggered disulfide bond reduction method to prepare hydrogels based on Fmoc-FF. Similar to our previous report on the design of the pro-gelator of Nap-FFE-ss-EERGD [50], we designed and synthesized a possible pro-gelator of Fmoc-FFE-ss-EE, which was shown in Figure 1. We believed that the pro-gelator could be converted by GSH to a gelator of Fmoc-FFE-s, resulting in the hydrogel formation. Similar to the gels of Fmoc-FF, we imaged that the resulting hydrogel of Fmoc-FFE-s would also be suitable for 3D cell culture. We used Fmoc-CS reported in our previous study and Fmoc-amino acids with side chain protected for solid phase peptide synthesis to produce the design compound. The pure compound was obtained by reverse phase high performance liquid chromatography (HPLC, Figures S1 and S2 in File S1).

### Hydrogel formation

The pro-gelator could be well solubilized in phosphate buffer saline (PBS, pH = 7.4) or cell culture Dul-becco's modified Eagle's medium (DMEM) medium with 10% of fetal bovine serum (FBS) at concentrations up to 5 wt% (50 mg/mL). We therefore tested its gelation ability by GSH conversion. As shown in Figure 1 (insert), a clear hydrogel could be obtained within 5 minutes after the addition of 4 equiv. of GSH to the DMEM solution with 10% of FBS of pro-gelator (0.5 wt%, 5 mg/mL). If using less amount of GSH, hydrogels could also form but it took a longer time. For example, for the PBS solution containing 0.5 wt% of the pro-gelator and 1 equiv. of GSH, it took about 50 minutes for the hydrogelation. The hydrogel was stable and would not change the clear appearance for more than six months at room temperature. The fast gelation kinetics, clear appearance, and good stability of the gel suggested its big possibility for 3D cell culture.

### Rheology

**Figure 2:**
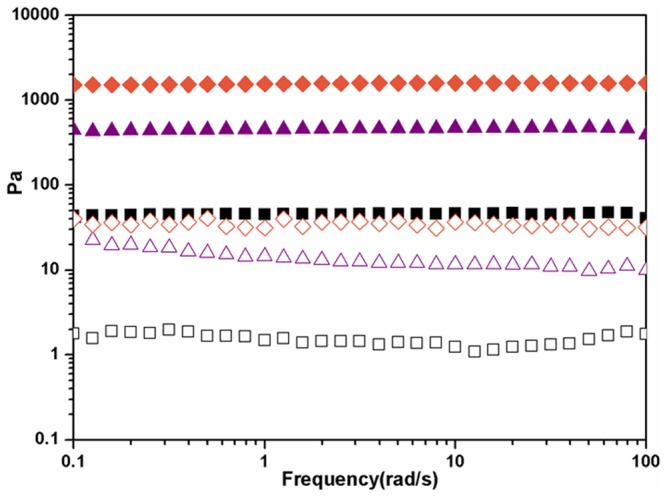
A representative rheological measurement with the mode of dynamic sweep at the frequency of 1 rad/s and the strain of 1% for a PBS solution containing 0.3 wt% of the pro-gelator and 4 equiv. of GSH (A) and rheological measurements with the mode of dynamic frequency sweep at the strain of 1% for gels from PBS solutions containing different concentrations of the pro-gelator (B) (filled symbols: G′ and open symbols: G″, diamonds: 0.3 wt%, triangles: 0.2 wt%, and squares: 0.1 wt%).

We used a rheometer to monitor the gelation process and characterize the resulting hydrogel (Figures S3, S4, S5, S6 in File S1). As shown in Figure 2A, the hydrogel formed instantly after the addition of GSH to a DMEM solution containing 0.5 wt% of the pro-gelator, as demonstrating by the value of elasticity (G′) dominating that of viscosity (G″). the mechanical property of the gel reached a balance after about 3,600 s, as indicating by the observation that both G′ and G″ reached plateaus. We then performed dynamic frequency sweep to characterize the mechanical property of hydrogels formed at 3,600 s with different concentrations of pro-gelator. As shown in Figure 2B, gels with higher concentration of the peptide obviously possessed bigger G′ values. For example, the G′ value of gel from 0.3, 0.2, and 0.1 wt% of the pro-gelator was about 1800, 450, and 45 Pa, respectively. The gels showed weak frequency dependences at the frequency range from 0.1 to 100 rad/s and the G′ value was at least 10 times bigger than its corresponding G″ value, suggesting the presence of high elastic networks in gels and the good mechanical property of the gels.

### Cryo-transmission electron microscopy (cryo-TEM)

**Figure 3:**
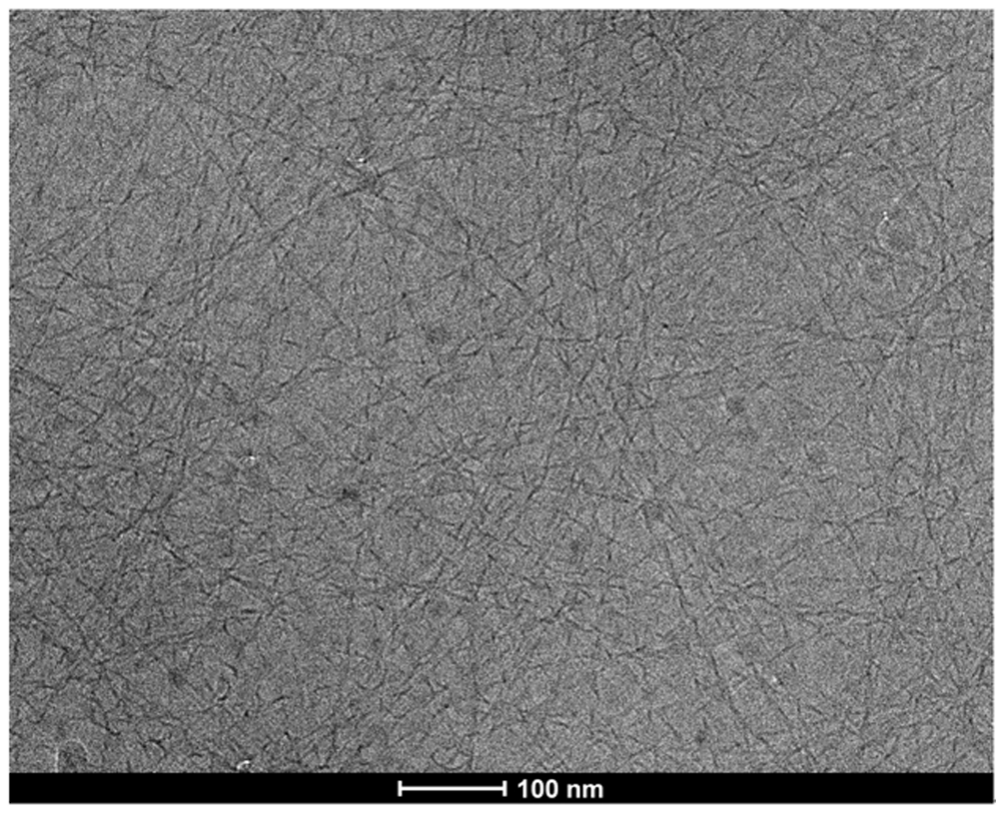
A cryo-transmission electron microscopy (cryo-TEM) image of the gel from a PBS solution containing 0.3 wt% of the pro-gelator with 4 equiv. of GSH.

We then obtained a cryo-transmission electron microscopy (cryo-TEM) image to characterize the self-assembled nano-structures in the gel. As shown in Figure 3, we observed uniform nanofibers with a width of about 8 nm in the gel. These fibers were helical with a left-handed helicity. They were longer than 2 µm and entangled with each other to form 3D networks for hydrogel formation. The examples of helical nanofibers in hydrogels were rare [51]–[55], and the helical nanofibers in this hydrogel might be useful as templates for the preparation of helical organic and in-organic nano-materials.

### Emission spectra

**Figure 4:**
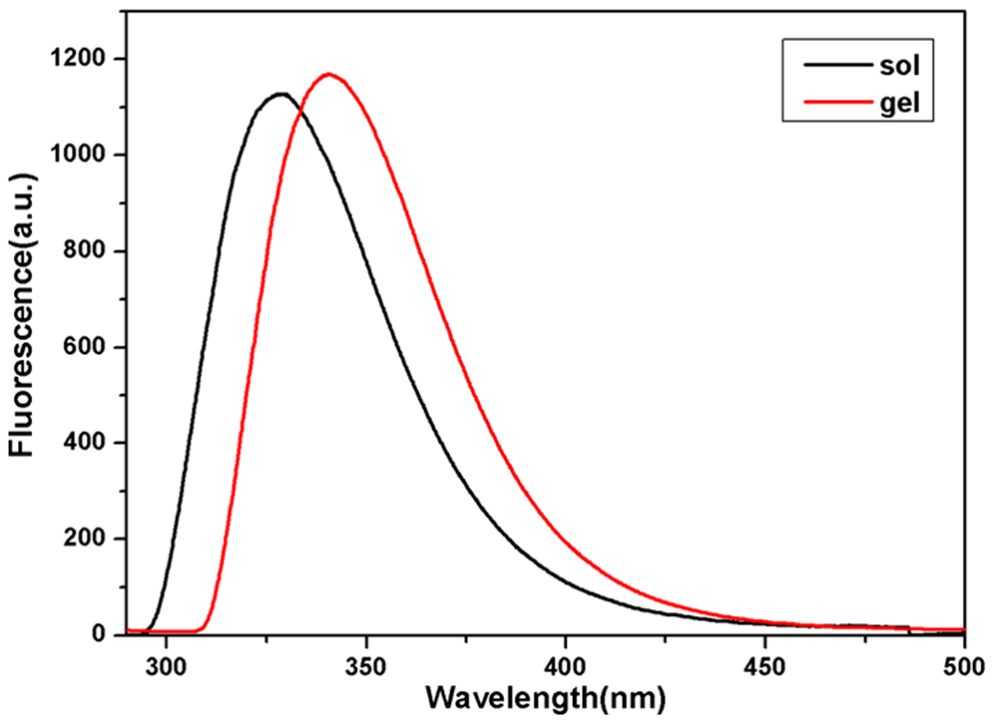
Emission spectra of PBS solution of the pro-gelator and the gel (excitation wavelength  = 265 nm).

In order to understand the aromatic arrangement of Fmoc in the nanofibers, we collected emission spectra of the PBS solution of pro-gelator and the resulting gel. As shown in Figure 4, the solution of pro-gelator exhibited a distinct peak centered at 328 nm, suggesting that the compound aggregated in the solution and the Fmoc groups favored the anti-parallel packing manner [35], [56]. This peak shifted to 342 nm after the gel formation, suggesting that the Fmoc groups overlapped in the parallel manner in the gel [35], [56]. The higher shoulder peak around 400 nm indicated that the Fmoc groups stacked more efficiently in the gel compared to those in the solution.

### Cell culture

**Figure 5:**
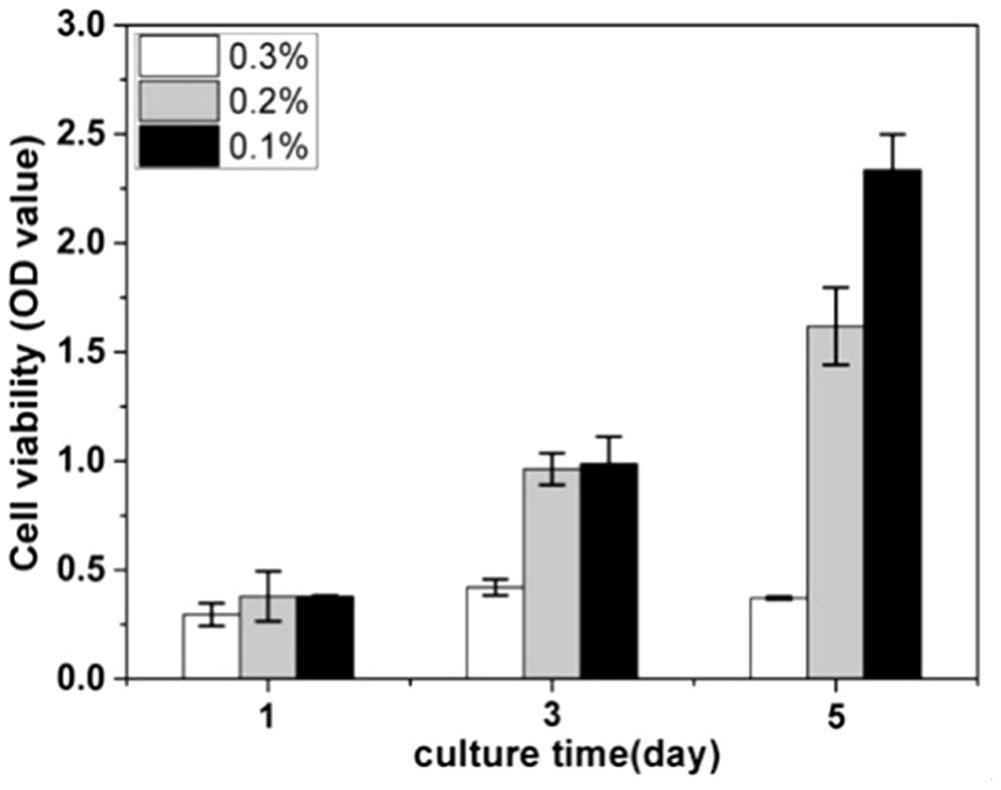
Cell proliferation rate of 3T3 cells in gels at different concentrations determined by the CCK-8 assay.

The gel from 0.3 wt% of the pro-gelator in PBS was stable for at least 5 days at 37°C in the incubator (Figure S7 in File S1), we therefore tested the possibility of resulting gels in 3D cell culture. We choose gels at different concentrations (0.3, 0.2, and 0.1 wt% of the pro-gelator) for 3D cell culture of NIH 3T3 cells. The cell-gel constructs could be obtained within 5 minutes after the addition of GSH. The volume of the gel was 50 µL in each well of the 96-well plate and the final cell density in the gel was 1,500,000/mL. 30 minutes after the formation of the cell-gel construct, we placed an additional 100 µL of DMEM supplemented with 10% of FBS on it. The CCK-8 assay was performed at day 1, 3, and 5 to determine the cell proliferation rate. Figure 5 showed that the 3T3 cells kept dividing in gels during the 5 culture days at the concentrations of 0.2 and 0.1 wt%, while they stopped proliferation in the gel at the concentration of 0.3 wt%. The gel from the solution of 0.1 wt% of the pro-gelator assisted the 3T3 cells the most efficiently within the three gels. The results in Figure S8 in File S1 indicated that the 3T3 cells at day 5 was homogeneously embedded in the gels formed by treating a DMEM solution containing 0.3 wt% of the pro-gelator with 4 equiv. of GSH. These observations indicated that gels with weaker mechanical properties were more suitable for the 3D culture of 3T3 cells, which was consistent with our previous results [46]. We hypothesized that cells in gels with weak mechanical properties could spread and divide more easily than those with strong mechanical properties.

**Figure 6:**
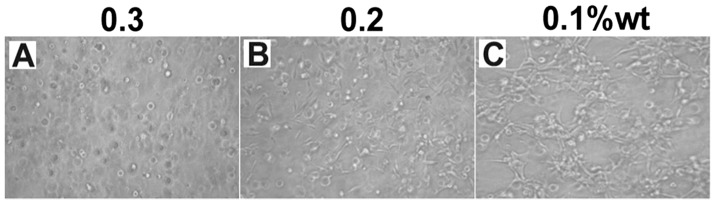
Optical images of cells at day 5 in gels at A) 0.3 wt%, B) 0.2 wt%, and C) 0.1 wt% (scale bars represent 10 µm).

After three days′ culture, we observed the cell morphology in gels by optical microscopy. As shown in Figure 6, 3T3 cells kept the round shape in the gel at 0.3 wt%. Most of cells spread well in the gel at 0.2 wt% and nearly total cells spread well in the gel at 0.1 wt%. These observations correlated well with the cell proliferation rate in Figure 5 and also suggested that gels with weaker mechanical property were more suitable for 3D cell culture of 3T3 cells. One shortcoming of our hydrogel is that we can not separate cells from gels post culture, and this problem may be addressed by developing other responsive supramolecular hydrogels.

## Conclusions

In conclusion, we have demonstrated that the disulfide bond reduction strategy can be applied to prepare supramolecular hydrogels based on Fmoc-FF. the resulting hydrogels are optical transparent and stable, which are suitable for 3D cell culture. The results show that weak gels are suitable for 3D culture of 3T3 cells. The concentration of GSH was in the range of 1–10 mM in biological system, and the one we used to prepare hydrogels in this study is 0.86–2.59 mM, suggesting that our method is compatible for 3D cell encapsulation and culture. The good versatility and ease of handle will endow its good possibility for cell culture, cell delivery, and control release of bioactive molecules.

## Materials and Methods

### Materials and General methods

All the starting materials were obtained from G. L. Biochem. (Shanghai) or Sangon Biotech. (Shanghai). Commercially available reagents were used without further purification, unless noted otherwise. All other chemicals were reagent grade or better. PBS buffer (0.01 M, pH 7.4) was prepared with pills purchased from Sangon Biotech. Co., Ltd. (Shanghai, China). Ultrapure water (18.2 MΩ. cm) was used throughout the experiment. HepG2 human liver cancer cells were supplied by the Molecular Biology Laboratory of Anhui Medical University. High resolution ESI/MS spectra were obtained on a GCT premier mass spectrometer (Waters). HPLC analyses were performed on an Agilent 1200 HPLC system equipped with a G1322A pump and in-line diode array UV detector using a YMC-Pack ODS-AM column with CH_3_OH (0.1% of TFA) and water (0.1% of TFA) as the eluent. ^1^H-NMR spectra were obtained on a 300 MHz Bruker AV300. Cells were routinely cultured in Dul-becco's modified Eagle's medium (DMEM, Hycolon) supplemented with 10% fetal bovine serum at 37°C, 5% CO_2_, and humid atmosphere.

### Solid phase peptide synthesis

The peptide derivative was prepared by solid phase peptide synthesis (SPPS) using 2-chlorotrityl chloride resin and the corresponding N-Fmoc protected amino acids with side chains properly protected by a tert-butyl group. The first amino acid was loaded on the resin at the C-terminal with the loading efficiency about 0.6 mmol/g. 20% piperidine in anhydrous N,N′-dimethylformamide (DMF) was used during deprotection of Fmoc group. Then the next Fmoc protected amino acid was coupled to the free amino group using O-(benzotriazol-1-yl)-N,N,N′,N′-tetramethyluroniumhexafluorophosphate (HBTU) as the coupling reagent and diisopropylethylamine (DIEA) as catalysis reagent. The growth of the peptide chain was according to the established Fmoc SPPS protocol. After the last amino acid was coupled, excessive reagents were removed by a single DMF wash for 5 min (5 mL per gram of resin), followed by 5 times DCM wash for 2 min (5 mL per gram of resin). The peptide was cleaved using 95% of trifluoroacetic acid (TFA) with 2.5% of trimethylsilane (TMS) and 2.5% of H_2_O for 30 min. TFA was removed by a rotary-evaporator, then 20 mL per gram of resin of ice-cold diethylether was added. The resulting precipitate was dissolved in DMSO directly for HPLC separation with MeOH containing 0.1% of TFA and H_2_O containing 0.1% TFA as eluents.

### Hydrogel formation

The stock solution of pro-gelator was prepared at the concentration of 10 mg/mL (1 wt%), and the stock solution of GSH was prepared at the concentration of 10.8 mg/mL. The molar concentration of GSH stock solution was 4 times that of pro-gelator stock solution. During the preparation of the above two stock solutions, Na_2_CO_3_ was used to adjust the final pH value to be 7.4. Mixing two stock solutions with equal volume and with different amounts of PBS solution resulted in solutions containing different concentrations of the pro-gelator. Gels would form after being kept at room temperature (22–25°C) for within 5 minutes. The same procedure was used to prepare gels in DMEM solution, but replacing PBS solution with the DMEM solution.

### CCK-8 assay

To quantify cell proliferation inside the cell-gel constructs, a CCK-8 assay was performed at a series of time points. A 3D Culture standard was made by encapsulating cells into hydrogels. Briefly, cells in culture medium with FBS, two stock solutions, and DMEM with FBS solution with different ratios were mixed together. The final cell density was 1,500,000/mL gel and the final concentration of the pro-gelator was 0.1, 0.2, or 0.3 wt%. The final solution was transferred to 96 well plates (50 µL per plate). After about half an hour, 100 µL of additional DMEM with 10% of FBS was added on the top of the gel. The plates were incubated in the 5% CO_2_ incubator. To perform the CCK-8 assay, each cell-gel construct was washed with DMEM twice, and then incubated with 100 µL of 10% (v/v) CCK-8 in serum-free DMEM. The plates were then incubated in the 5% CO_2_ incubator for 4 h at 37°C. The absorbance at 450 nm was determined using the microplate reader (MultiskaniMark, Bio-Rad, USA). The experiments were conducted for three times and SD was determined.

## Supporting Information

File S1 **Synthesis and characterization, rheology, optical images, and Z-stacking scanning imaging in confocal microscope to show the homegeneous distribution of cells are included in File S1.**
(DOC)
