# Supplementary material for: Glutathione-Triggered Formation of a Fmoc-Protected Short Peptide-Based Supramolecular Hydrogel
Source: PLoS One. 2014 Sep 15;9(9):e106968. doi: 10.1371/journal.pone.0106968 (PMC4164459; doi:10.1371/journal.pone.0106968)
Supplement: File S1 — Synthesis and characterization, rheology, optical images, and Z-stacking scanning imaging in confocal microscope to show the homegeneous distribution of cells are included in File S1. (DOC) [file pone.0106968.s001.doc]

**Supporting Information**

**Characterization of the pro-gelator:** ^1^H NMR (300 MHz, DMSO-d_6_) δ8.25-8.09 (m, 3H), 8.08-7.90 (m, 3H), 7.89-7.81 (d, 2H), 7.64-7.53 (t, 3H), 7.43-7.33 (m, 3H), 7.32-7.27 (t, 2H), 7.26-7.16 (m, 8H), 7.15-7.06 (m, 2H), 4.30-4.19 (m, 3H), 4.17-4.13 (m, 1H), 4.13-4.05 (m, 2H), 3.93 (s, 1H), 3.61-3.52 (t, 2H), 3.09-3.01 (m, 1H), 2.97-2.81 (m, 2H), 2.79-2.65 (m, 5H), 2.37-2.29 (m, 4H), 2.28-2.20 (m, 5H), 2.01-1.83 (m, 4H), 1.82-1.65 (m, 3H). HR-MS: calc. M = 1155.39, obsvd. [M+H]^+^ = 1156.4000.

***Figure S1*.** ^1^H NMR of Fmoc-FFE-ss-EE


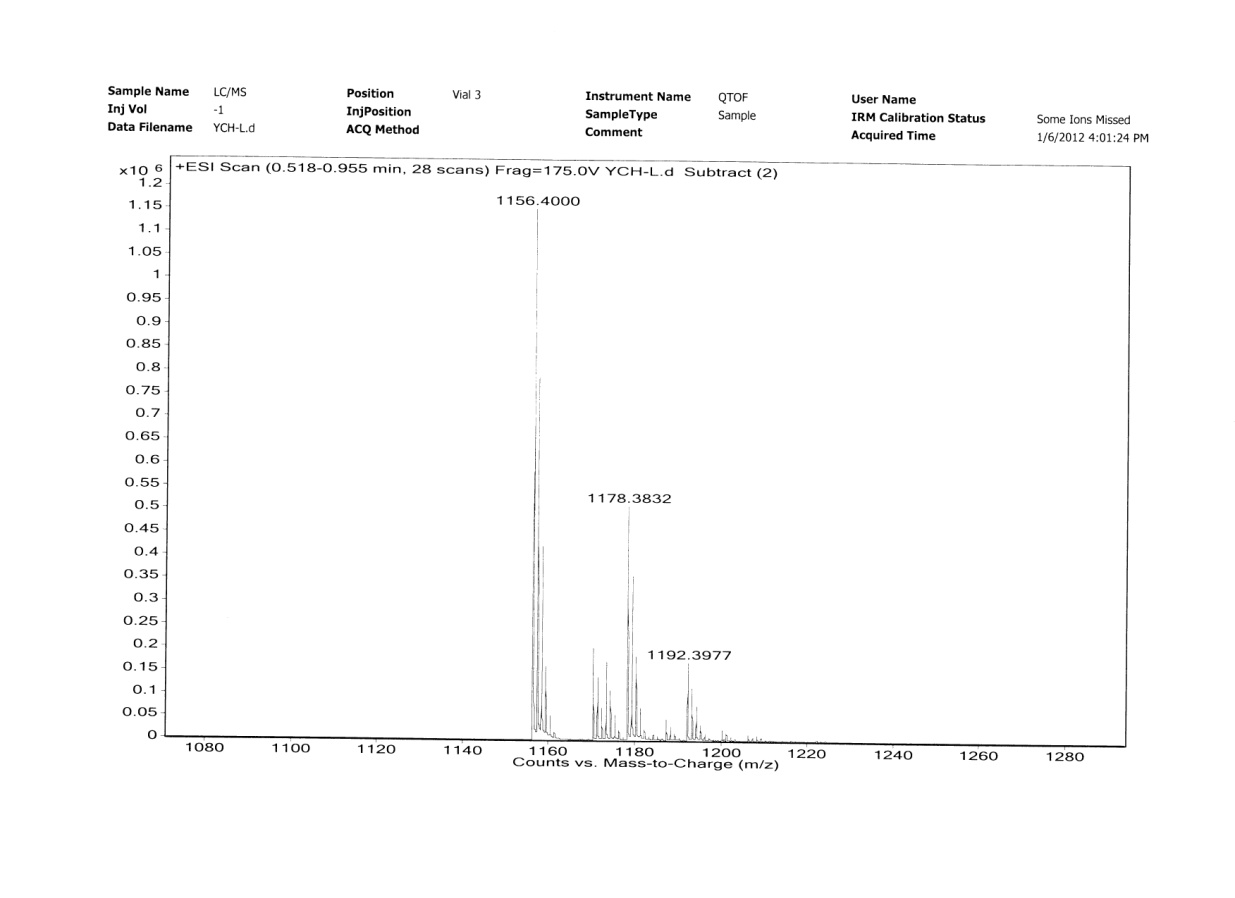


***Figure S2*.** HR-MS of Fmoc-FFE-ss-EE


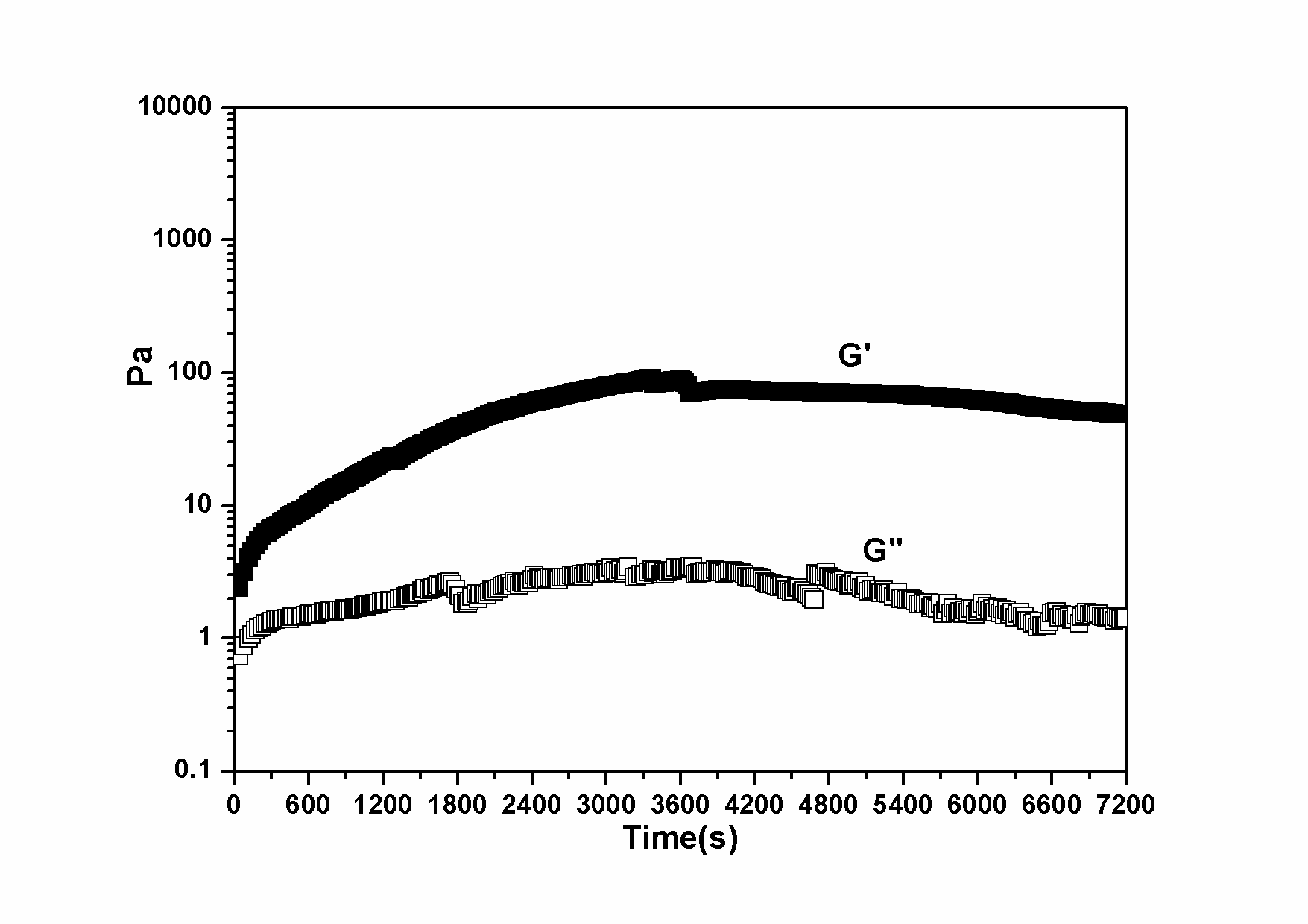


***Figure S3*.** Dynamic time sweep at the strain of 1% for the gel from solution containing 0.1 wt% of the pro-gelator


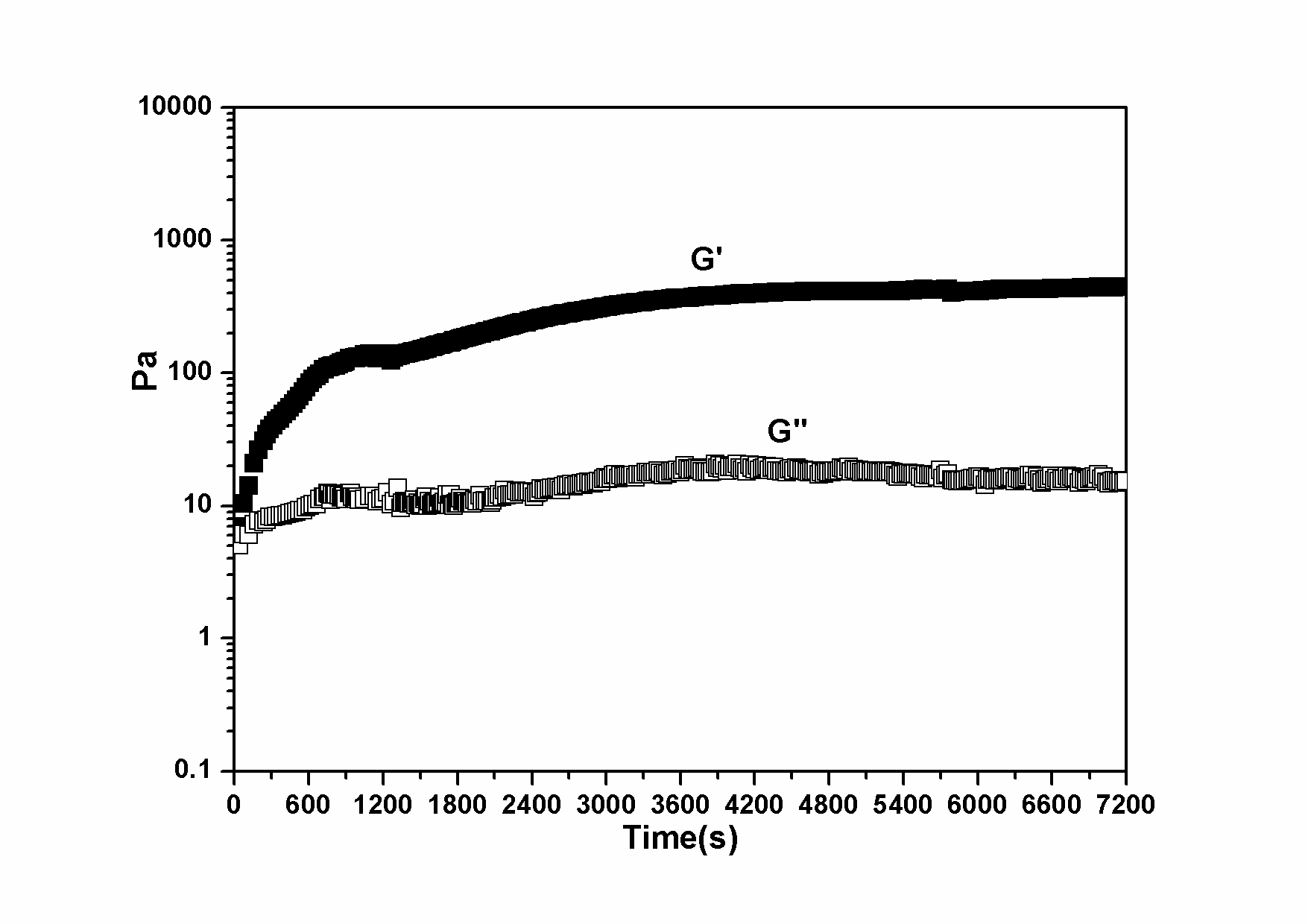


***Figure S4*.** Dynamic time sweep at the strain of 1% for the gel from solution containing 0.2 wt% of the pro-gelator


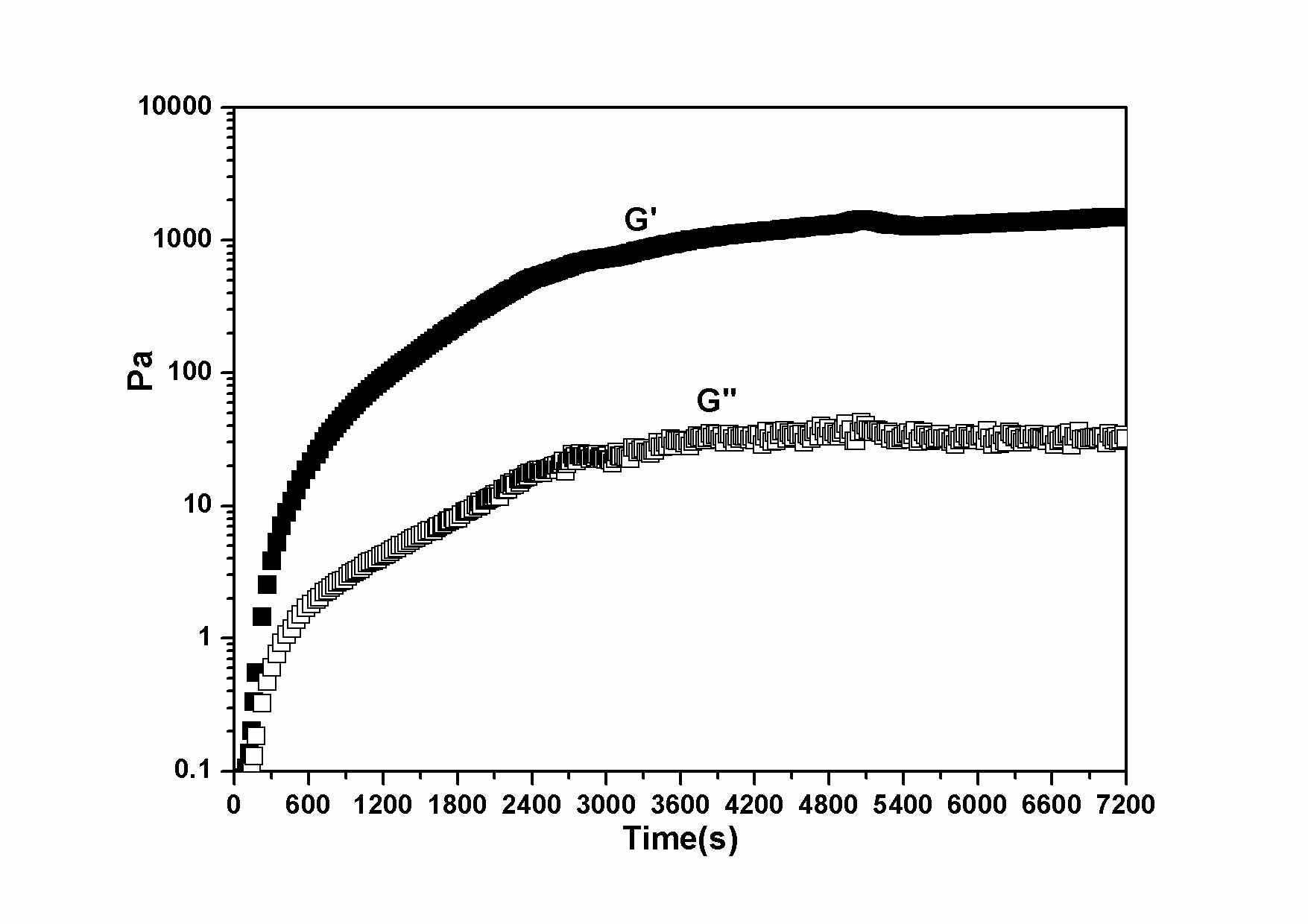


***Figure S5*.** Dynamic time sweep at the strain of 1% for the gel from solution containing 0.3 wt% of the pro-gelator


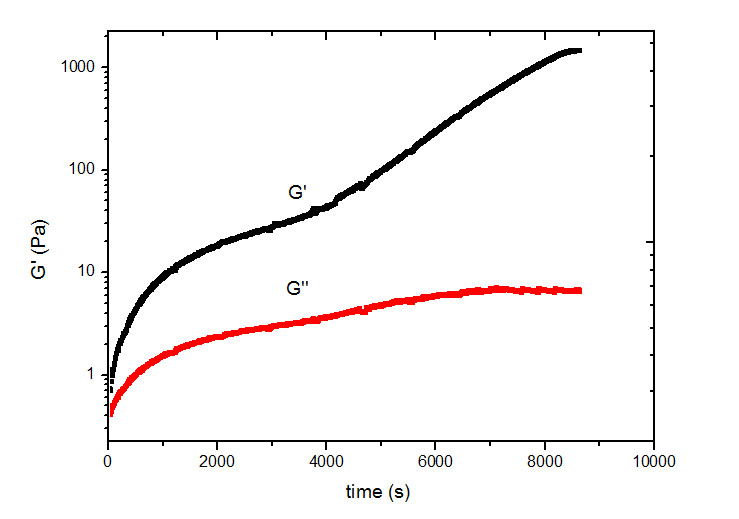


***Figure S6.*** Dynamic time sweep (with incubation time) at the strain of 1% for the gel from solution containing 0.3 wt% 1 equiv. of GSH of the pro-gelator


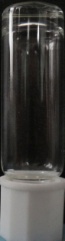

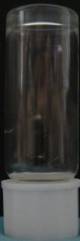

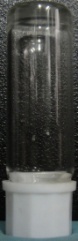

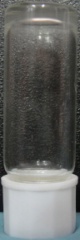


***Figure S7.*** Optical images of gels from 0.3 wt% of the pro-gelator in PBS at 37 ^0^C in the incubator at different times (from left to right: day 0, day 1, day 4, and day 5. the gel at day 5 changed to slightly opaque due to water evaporation).

*
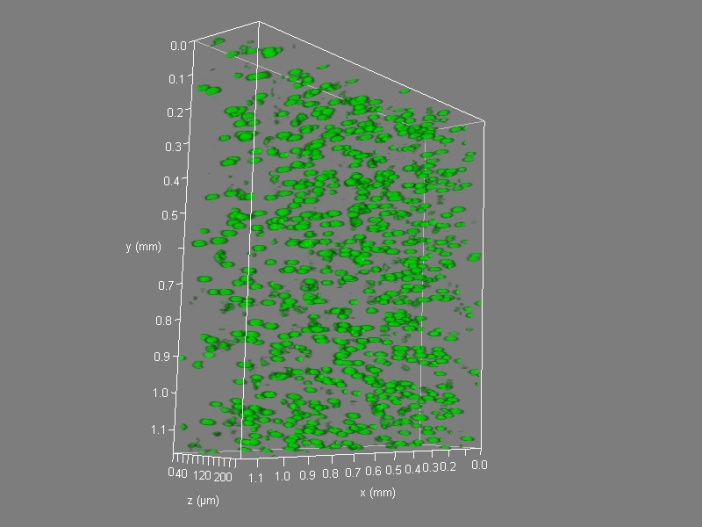
*

***Figure S8.*** Z-stacking scanning in confocal microscope indicated that the 3T3 cells was homogeneously embedded inside the gel formed by treating a DMEM solution containing 0.3 wt% of the pro-gelator with 4 equiv. of GSH.
